# Identifying the Severity of Dementia Based on Cognitive Performance and Index of Independence in Basic Activities of Daily Living

**DOI:** 10.1002/alz.091868

**Published:** 2025-01-03

**Authors:** Duong Huynh, Reza Hosseini Ghomi, Bin Huang, Mary Patterson

**Affiliations:** ^1^ BrainCheck, Inc, Austin, TX USA; ^2^ Frontier Psychiatry, Billings, MT USA; ^3^ BrainCheck, Austin, TX USA

## Abstract

**Background:**

Alzheimer’s disease and related dementias (ADRD) are characterized by impairments in an individual’s cognitive and functional abilities. Computerized cognitive tests have been shown to be effective in detecting mild cognitive impairment and ADRD but whether they can stage the ADRD disease remains to be studied.

**Method:**

Retrospective analysis was performed on a BrainCheck dataset containing 1,749 ADRD patients with different cognitive and functional assessments completed for cognitive care planning, including the Dementia Severity Rating Scale (DSRS), the Katz Index of Independence in Activities of Daily Living (ADL), and the BrainCheck cognitive assessment battery (BC‐Assess). The patients were staged according to their DSRS total score (DSRS‐TS): 971 mild‐stage (DSRS‐TS: 10‐18), 664 moderate‐stage (19‐26), and 114 severe‐stage (37‐54) patients. Pearson correlation was calculated to assess the strength of associations between BC‐Assess overall score (BC‐OS), ADL total score (ADL‐TS), and DSRS‐TS. A three‐way Multivariate‐Analysis‐of‐Variance (MANOVA) was used to examine how the ADL‐TS and the BC‐OS combined differ across stages after controlling for age group and gender. Multinomial logistic regression was used to evaluate the possibility of using patients’ ADL‐TS and/or BC‐OS to predict their condition.

**Result:**

Correlations between scores are moderate. A stronger correlation was found between the DSRS‐TS and the ADL‐TS (r = ‐0.58) than that between the DSRS‐TS and the BC‐OS (r = ‐0.54) and between the ADL‐TS and the BC‐OS (r = 0.38). The ADL‐TS and BC‐OS vary significantly across ADRD stages. ROC analysis of the logistic regression shows that, although either ADL‐TS or BC‐OS can effectively predict ADRD stage, highest accuracy is when both scores are included in the model (ROC‐AUC: mild = 0.94; moderate = 0.77; severe = 0.9; micro‐average = 0.93). Except a low sensitivity in identifying the moderate group, a sensitivity/specificity of at least 76% was observed for all models and stages.

**Conclusion:**

Our results suggest a progressive decline in both cognitive and functional abilities as patients move through the stages of ADRD. Among ADRD patients, performance in a cognitive assessment (BC‐Assess) or in basic activities of daily living (ADL) is informative of severity diagnosis. However, combining both measures generates highest accuracy.

